# *pry-1* interacts with *bar-1* to regulate *vit-2* expression, lipid levels, and lifespan in *Caenorhabditis elegans* adults

**DOI:** 10.17912/micropub.biology.000987

**Published:** 2023-10-20

**Authors:** Atreyee De, Bhagwati Gupta

**Affiliations:** 1 Biology, McMaster University, Hamilton, Ontario, Canada

## Abstract

The *C. elegans* Axin homolog, PRY-1 , is essential for multiple biological processes including vulval development, lipid metabolism, and lifespan maintenance. *pry-1* mutants exhibit lower lipid contents and knockdowns of *vit* genes in *pry-1* mutants can restore lipid levels, implicating vitellogenins’ involvement in PRY-1 -mediated lipid homeostasis. As a component of the canonical WNT signal transduction pathway, PRY-1 inhibits the function of the β-catenin ortholog BAR-1 during vulval development and other developmental events. We showed earlier that a constitutively active form of BAR-1 causes a reduction in lipid contents, however, whether PRY-1 interacts with BAR-1 to regulate lipid levels and other processes is unknown. To this end, we examined the phenotypes of *pry-1* and *bar-1* single and double mutants. Our data suggest that the *pry-1 - bar-1* genetic pathway regulates *vit-2* expression, lipid homeostasis, and the lifespan of animals.

**Figure 1. bar-1(ga80) suppresses pry-1(gk3682) phenotype involving vit-2 transcription, lipid levels, vulval development, and lifespan:**
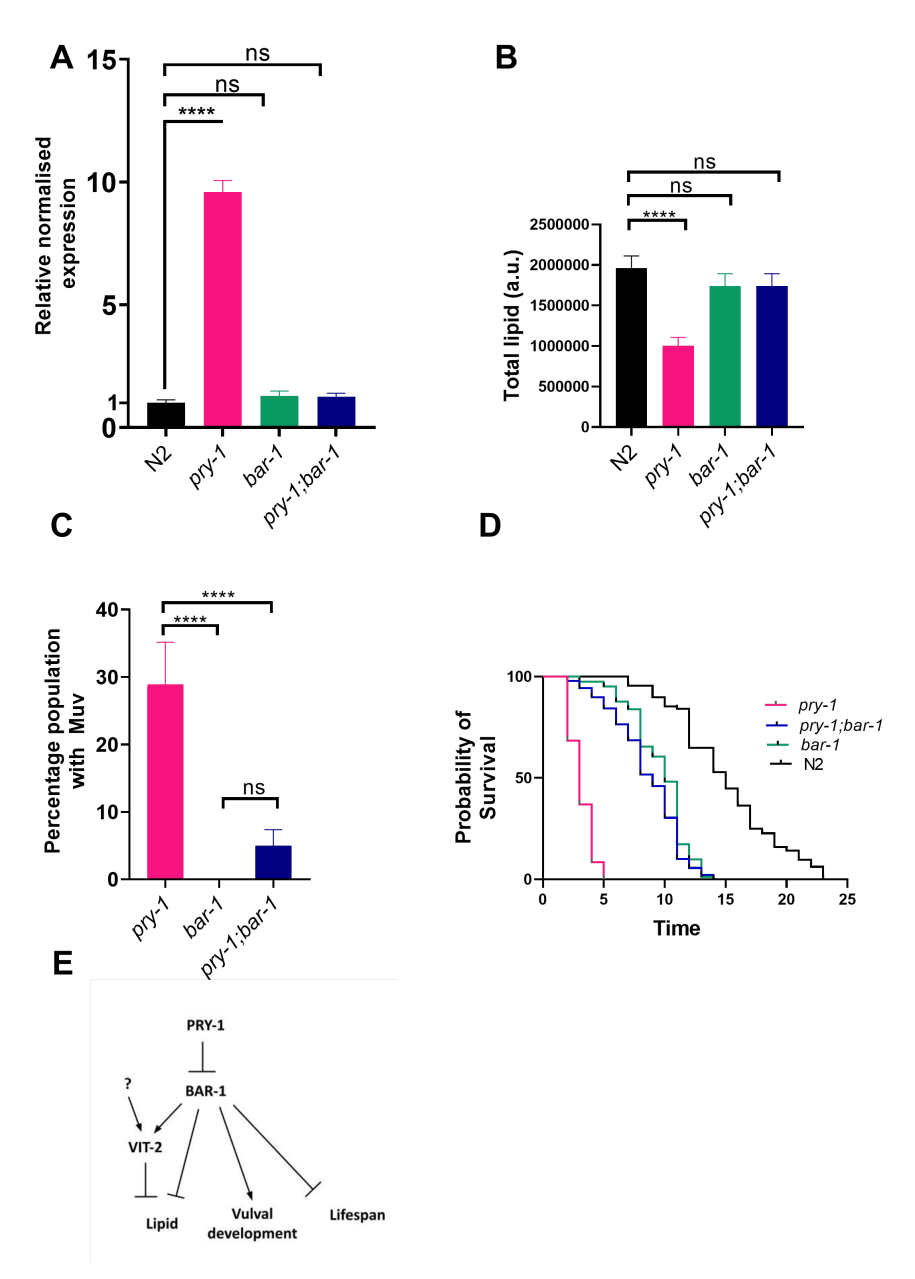
(A) qRT-PCR analysis of *vit-2* in day-1 adults of *pry-1(gk3682), bar-1(ga80)* , and *pry-1(gk3682);bar-1* (ga80) animals. The relative normalized expression has been plotted. (B) Quantification of Oil Red O signal in mutant worms. (C) Muv phenotype in adults. (D) Lifespan analysis of animals. (E) A model of *pry-1* and *bar-1* function based on results presented in this study. In addition to *bar-1* , *vit-2* expression may be regulated by other factors. Our genetic analysis suggests that the *pry-1-bar-1* pathway regulates lipid levels via both *vit-2* -dependent and -independent manners. Histograms in A, B, and C show the means of at least three replicates and the error bars represent the standard error of the mean (SEM). The lifespan data in D is based on three independent batches. The p values are represented by stars (**** p<0.0001). The mean lifespan of N2 was 15.1±0.3 days, *pry-1(gk3682)* 3.1±0.9 days, *bar-1(ga80)* 10±0.2 days, and *pry-1(gk3682); bar-1(ga80)* 8.9±0.3 days. p <0.0001 for *pry-1* vs. N2, p<0.0001 for *bar-1* vs. N2, p<0.0001 for *pry-1; bar-1* double vs. *pry-1* , and p = 0.02 for *pry-1; bar-1* double vs. *bar-1*

## Description

In a previous study, we demonstrated that *vit-2* is the only vitellogenin that is upregulated in *pry-1 (gk3682)* day-1 young adults (Mallick & Gupta, 2020) . While the levels of *vit-2* decline slightly in day-4 old adults, the trend remains the same, suggesting that *pry-1* negatively regulates *vit-2* expression. Since a key effector of PRY-1 -mediated WNT signaling is the β-catenin BAR-1 , we investigated whether the *pry-1 - bar-1* pathway regulates *vit-2* .

To this end, a *pry-1 (gk3682); bar-1 ( ga80 )* double mutant strain was generated. The *vit-2* levels in these animals and *bar-1 ( ga80 )* animals were comparable to the wildtype N2 ( Figure 1A ), leading us to conclude that *bar-1* acts downstream of *pry-1* to regulate *vit-2* expression. Furthermore, since *vit-2* expression in *bar-1 ( ga80 )* strain was unaffected, it suggests that other genes act redundantly with *bar-1* to affect *vit-2* .

We also examined lipid levels in the *pry-1 ; bar-1* double mutant using Oil red O staining. Our lab had previously reported that *pry-1* mutants have significantly lower lipid stores (Mallick & Gupta, 2020; Ranawade et al., 2018) . Interestingly, both *bar-1 ( ga80 )* and *pry-1 (gk3682); bar-1 ( ga80 )* animals showed lipid levels significantly higher than *pry-1* mutant and comparable to the wildtype N2 ( Figure 1B ). Thus, like *vit-2* regulation, *pry-1* appears to act through *bar-1* to maintain lipid levels.

Next, we investigated the lifespan phenotype of mutant animals. Previously, *pry-1* and *bar-1* were shown to affect aging (Mallick et al., 2022; Essers et al. 2005) , but the interaction between the two genes was not examined. We found that *pry-1 (gk3682)* and *bar-1 ( ga80 )* animals were short-lived with mean lifespans of 3.1 days and 10 days, respectively ( Figure 1D ). The examination of *pry-1 (gk3682); bar-1 ( ga80 )* double mutants revealed a mean lifespan of 8.9 days, which was comparable to *bar-1 ( ga80 )* alone but significantly longer than *pry-1 (gk3682)* ( Figure 1D ). Thus, the lifespan defect of animals lacking *pry-1* function is suppressed by mutations in *bar-1* .

The above epistasis relationship between *pry-1* and *bar-1* is similar to what has been reported in the case of vulval development, where both genes act in a canonical WNT pathway to regulate vulval precursor cell induction (Gleason et al., 2002; Sheetharaman et al., 2010) . Hence, while *pry-1* mutants show a multivulva (Muv) phenotype (Gleason et al., 2002) , *bar-1* mutants are vulvaless (Vul) and suppress the Muv phenotype in *pry-1* mutants (Eisenmann, et al., 2000; Gleason et al., 2002) . As expected, the Muv phenotype of *pry-1 (gk3682)* was strongly suppressed in double mutants ( Figure 1C ).

The *pry-1 ,* and *bar-1* mutant adults frequently show a protruding vulva (Pvl) morphology (Eisenmann et al., 1998, Eisenmann, et al., 2000, Gleason et al., 2002) , a phenotype that may arise from defects in vulval induction, vulval morphogenesis, or lateral seam cells (Schindler & Sherwood, 2013) . We quantified the defect in single and double mutants (see Methods): *bar-1 ( ga80 )* 54% Pvl, *pry-1 (gk3682)* 62% Pvl; and *pry-1 (gk3682); bar-1 ( ga80 )* 71% Pvl. While this suggests that *bar-1 ( ga80 )* does not suppress *pry-1 (gk3682)* phenotype, it is important to point out that the precise reason for the Pvl phenotype in *pry-1* mutants is unknown, making it difficult to draw a firm conclusion from the data.

In conclusion, our findings demonstrate that in addition to vulval development, the *pry-1 - bar-1* genetic pathway regulates *vit-2* expression, lipid homeostasis, and the lifespan of animals.

## Methods

**Strain and growth conditions**

Worms were grown at 20°C on standard nematode growth media plates seeded with *E. coli* OP50 . The strains used in this study are: N2 (wildtype), VC3710: *pry-1 (gk3682)* , DY788: *bar-1 ( ga80 )* , and DY765: *pry-1 (gk3682); bar-1 ( ga80 )* .

**Lifespan analysis**

Lifespan experiments were performed at 20°C using the protocol described previously (Murphy et al. 2003, Amrit et al. 2014 ). Each batch contained 30-50 synchronized day-1 adults.

**Oil Red O staining**

Oil Red O staining was performed as previously reported (Ranawade et al. 2018) . Briefly, animals at day-1 adulthood were collected after washing with 1X PBS buffer from the plate and treated as described in the protocol. Animals were mounted and imaged with Q imaging software and Micropublisher 3.3 RTV color camera outfitted with DIC optics on a Nikon 80i microscope. NIH ImageJ software was used to quantify Oil Red O intensities (Soukas et al. 2009) . 15 to 30 day-1 adults were randomly selected from each genotype in at least three separate batches.

**Vulval morphology**

Muv and Pvl phenotypes were quantified in day-1 adults using a stereomicroscope. Animals were examined in three batches, each batch contained 30-50 animals. The Muv hermaphrodites show multiple pseudo vulvae in the ventral region due to ectopic induction of vulval precursor cells. The Pvl defect was classified as mild or severe based on the approximate size of protruding vulvae. The *bar-1 ( ga80 )* animals showed 24% mild Pvl and 30% severe Pvl phenotypes (n = 151). The *pry-1 (gk3682)* and *pry-1 (gk3682); bar-1 ( ga80 )* animals were 62% Pvl (all severe, n = 91) and 71% Pvl (all severe, n = 243), respectively. The p values for Pvl analysis are p <0.0001 for *pry-1* vs *bar-1* , p <0.0001 for *bar-1* vs *pry-1 ; bar-1* , p = 0.1742 for *pry-1* vs *pry-1 ; bar-1* .

**qPCR analysis**

Total RNA was extracted from bleached synchronized day-1 adults using the Tri-reagent (Catalog Number T9424, Sigma-Aldrich Canada) according to manufacturer’s instructions. The RNA was used to synthesize cDNA as per the instructions provided by the SensiFAST™ cDNA kit (Catalog Number BIO-65054, USA). Quantitative real-time PCR (qRT-PCR) analysis was performed using a CFX 96 BioRad cycler in triplicate with SensiFAST™ SYBR® Green Kit (Catalog Number BIO-98005, USA), according to the manufacturer’s instructions. Primers used in the study are listed below:

*iscu-1* FP: TCAGCCGCGAAAACACTCCTG and RP: ACATTTCGCGGGTTCTCGTAGTG

*vit-2* FP: GACACCGAGCTCATCCGCCCA and RP: TTCCTTCTCTCCATTGACCT

**Statistical analysis**

GraphPad Prism 9.5.1 was used to plot all the graphs and perform statistical analysis. Data arising from multiple experiments were combined and subjected to joint analysis. For multiple comparisons, an analysis of variance (ANOVA) test was conducted. Graphs were plotted with SEM. For Pvl comparison. Chi-square test was done. qRT-PCR results were analyzed using CFX Maestro 3.1 software (Bio-Rad, Canada; https://www.bio-rad.com/en-ca/product/cfx-maestro-software-for-cfx-real-time-pcrinstruments ). SigmaPlot 14 was used to calculate the mean lifespan using the log-rank (Kaplan-Meier) method.
